# The Induction of Dissociative States: A Meta-Analysis

**DOI:** 10.1016/j.bpsgos.2025.100521

**Published:** 2025-04-28

**Authors:** Benjamin Brake, Lillian Wieder, Natasha Hughes, Ivonne Saravia Lalinde, Danielle Marr, Dali Geagea, Susannah Pick, Antje A.T.S. Reinders, Sunjeev K. Kamboj, Trevor Thompson, Devin B. Terhune

**Affiliations:** a Department of Neuroscience, Institute of Psychiatry, Psychology and Neuroscience, King’s College London, London, United Kingdom; b Department of Psychology, Institute of Psychiatry, Psychology and Neuroscience, King’s College London, London, United Kingdom; c Department of Clinical Psychology, Leiden University, Leiden, the Netherlands; d Department of Psychosis Studies, Institute of Psychiatry, Psychology and Neuroscience, King’s College London, London, United Kingdom; e Institute for Psychology, Friedrich Alexander Universität Erlangen-Nuremberg, Erlangen, Germany; f Centre for Children’s Burns and Trauma Research, The University of Queensland, Brisbane, Queensland, Australia; g Department of Psychological Medicine, Institute of Psychiatry, Psychology and Neuroscience, King’s College London, London, United Kingdom; h Clinical Psychopharmacology Unit, Department of Clinical, Educational and Health Psychology, University College London, London, United Kingdom; i School of Human Sciences, University of Greenwich, London, United Kingdom

**Keywords:** Dissociative, Ketamine, Mirror gazing, NMDAR, PTSD, Psychedelics

## Abstract

**Background:**

Dissociative states, characterized by discontinuities in awareness and perception, occur in a diverse array of psychiatric disorders and contexts. These states have been experimentally modeled in the laboratory through various induction methods, but relatively little is known about the efficacy and comparability of different experimental methods.

**Methods:**

In this meta-analysis, we quantified dissociative states, as indexed by a standardized instrument (Clinician-Administered Dissociative States Scale), at baseline in varied diagnostic categories and in response to different experimental induction methods (psychological techniques and pharmacological agents) in both clinical and nonclinical samples. Primary outcomes were state dissociation effect sizes (Hedges’ *g*) (PROSPERO registration: CRD42022384886). A total of 2214 articles were screened, which yielded 123 eligible articles and 155 effect sizes comprising 6692 individuals.

**Results:**

High levels of baseline state dissociation were observed in multiple diagnostic categories relative to controls, with the largest effects found in the dissociative and complex subtypes of posttraumatic stress disorder (PTSD-DC). In controlled experiments, induced state dissociation was most pronounced in response to mirror gazing and multiple pharmacological agents, with effects in ketamine and cannabis exceeding baseline state dissociation in PTSD-DC. The effect sizes were characterized by pronounced heterogeneity but were not reliably associated with methodological features of the original studies.

**Conclusions:**

Elevated state dissociation is present in multiple diagnostic categories, and comparable or higher levels can be reliably induced in controlled experiments using psychological techniques and pharmacological agents. These results demonstrate the efficacy of several methods for experimentally modeling dissociation and have implications for measuring adverse events and predicting outcomes in clinical interventions that involve pharmacological agents.

## Plain Language Summary

We conducted a meta-analysis of studies that measured state dissociation (e.g., feeling detached from one’s environment) in different clinical groups and in response to different procedures. State dissociation was most pronounced in posttraumatic stress disorder, with comparable dissociative effects found in response to mirror gazing and ketamine. These results demonstrate that clinically significant levels of dissociation can be reliably induced using a variety of methods and have implications for attempts to experimentally study dissociation in controlled settings.

Dissociation comprises a constellation of symptoms characterized by discontinuities in awareness, volition, and perception (1,2). These experiences range from episodes of depersonalization and derealization, encompassing feelings of detachment from emotional or bodily states and/or one’s environment, respectively, to distortions in control, identity, and memory. Dissociation is increasingly being recognized as a transdiagnostic symptom prevalent in a wide variety of psychiatric conditions (2). Elevated levels of dissociation may also serve as a salient marker of clinical outcomes including a higher burden of illness (3), poorer quality of life (4), more pronounced symptomatology (5, 6, 7), and poorer treatment outcomes (8).

The clinical significance of dissociation underscores the need for controlled research on these symptoms, but no consensus experimental model of dissociation exists. Psychological techniques range from those that induce dissociative states through modulation of awareness and perception (e.g., mirror gazing) or exposure to stressors (9). Multiple pharmacological agents have been shown to trigger dissociation, particularly those that function as NMDA receptor (NMDAR) antagonists, such as ketamine and nitrous oxide (N_2_O) (10). To our knowledge, there has not yet been any attempt to quantitatively synthesize and contrast these different induction effects or compare them against baseline dissociative states in diagnostic categories.

A robust experimental model of dissociative states would offer novel opportunities for identifying neurophysiological and neurochemical markers of dissociative states, elucidating the impact of dissociation on other symptoms (e.g., hallucinations), and could inform both the diagnosis and treatment of a range of psychiatric conditions (10,11). Moreover, because NMDAR antagonists and serotonergic psychedelics are used, or have been proposed, as mainstream antidepressants (12), studying their dissociative effects may aid in advancing understanding of treatment-related adverse events (13) and treatment outcomes (14), which often covary with dissociative responses.

In this meta-analysis, we sought to fill outstanding gaps in current knowledge regarding the experimental induction of dissociative states and their comparability to baseline dissociation in diagnostic categories. As in other meta-analyses (2), we sought to increase uniformity of comparisons within and across categories, and thus we restricted our analyses to studies that measured dissociative states using the Clinician-Administered Dissociative States Scale (CADSS) (15), the most widely used measure of state dissociation (16). Our primary aims were to quantitatively synthesize and compare baseline state dissociation effects in different diagnostic categories and induced state dissociation effects in response to different psychological techniques and pharmacological agents. Our secondary aims were to explore the factors that moderate the magnitude of state dissociation effects within and across categories.

## Methods and Materials

This preregistered study (http://t.ly/I-ppg) was conducted under the updated Preferred Reporting Items for Systematic Reviews and Meta-Analyses (PRISMA) 2020 guidelines (17).

### Eligibility Criteria

The inclusion criteria were English language, full article in a peer-reviewed journal, participants ages ≥18 years, and inclusion of descriptive statistics and sample sizes for the CADSS in a diagnostic group and a nonclinical control group or in an experimental and control condition. Exclusion criteria included reviews, abstracts, dissertations, or case studies; data overlapping with included studies; use of a dissociation-attenuating agent; and CADSS completion after an extended period (>12 hours).

### Search Strategy

In October 2022, 2 researchers (BB and LW) independently searched MEDLINE, PubMed, PsycINFO, and Embase using terms related to the CADSS (see the Supplement). The search was limited to studies published since 1998, the initial publication year of CADSS. All eligible studies were integrated into a database using Covidence (Veritas Health Innovation; http://www.covidence.org). The search was repeated in June 2023 and March 2024 and yielded 6 and 4 additional studies, respectively.

### Study Selection

Two independent raters (BB, DG, NH, DM, ISL, LW) independently screened and assessed all studies for eligibility using a 2-stage procedure. First, they screened titles and abstracts, rejecting articles that did not meet eligibility criteria. Then, they reviewed the remaining articles to finalize the study list. A third reviewer (DBT) resolved discrepancies at either stage. If eligible articles lacked sufficient CADSS data, corresponding authors were contacted via email (up to 3 attempts over 3 months).

### Data Extraction

Data extraction was performed by 2 raters (BB, DG, NH, DM, ISL, LW). The primary outcomes extracted were CADSS scores (15) in a target condition/group and a control condition/group. Secondary outcomes included CADSS subscale scores and correlations between trait dissociation scores and CADSS scores. Both raters independently extracted and coded data using a prepiloted extraction form in Covidence that covered study details (authors, title, journal, publication date, country); demographics (sample size, gender distributions, age, education, ethnicity); study design (repeated-measures, between-groups, mixed-model); category (diagnostic group, psychological technique, pharmacological agent); CADSS information (administrator [clinician/experimenter vs. self-report], mode of administration [in person or remote], version [number of items], number of measurement timepoints, subscales, language); trait dissociation measure; clinical study methods (diagnosis, diagnostic criteria, diagnostic method, comorbidities, control type [healthy or clinical], clinical control diagnosis); pharmacological study methods (CADSS measurement times, drug class, dose, administration method and duration, concurrent drug use information, active/inert placebo information); psychological technique (method, control condition/group information); other methodological details (counterbalancing, inclusion of suggestion for dissociation); descriptive statistics for CADSS scores (total and subscales in all conditions); and correlations between trait dissociation and CADSS scores. If descriptive statistics were not reported, they were extracted from figures using WebPlotDigitizer (version 4.6; https://automeris.io/) when possible. Discrepancies were resolved with a third reviewer and sometimes a fourth. Overall, there was 91% agreement between raters (range: 85%–98%).

### Methodological Quality

Two raters independently assessed the quality of each study using a 15-item scale (see the Supplement) concerning study objectives, participant recruitment, demographic data, inclusion/exclusion criteria, clarity of procedure, blinding, preregistration, and relative matching of groups/conditions. The items, adapted from a previous meta-analysis (18), were based on Cochrane criteria and PRISMA recommendations (19). Each item was rated on a categorical scale (0 = criterion not met, 1 = met), and a percentage met total was computed for each study; DBT resolved discrepancies. There was 90% agreement between raters (range: 63%–100%; mean kappa = 0.80; range: 0.25–1).

### Meta-Analysis and Meta-Regression

Descriptive statistics (means, standard deviations [SDs], and *n*s [sample sizes] or other suitable statistics) were used to compute Hedges’ *g*s (and standard errors [SEs]) for inclusion in random effects meta-analyses when there were 3 or more effect sizes per category (Supplemental Methods). Multiple effect sizes were extracted and included in meta-analyses for specific categories only when data from distinct samples were reported (see Table S2). For studies that reported multiple time points for the same participants (e.g., ketamine studies), we included only the effect size that corresponded to the peak response. Some categories had an insufficient number of effect sizes and were thus consolidated into higher-order categories. Categories included different diagnostic categories, psychological techniques, and pharmacological agents. For each category, we computed standardized mean differences (SMDs) and 95% CIs after outlier removal [studentized residuals > |3.3| (20)]. Although analysis of raw CADSS scores could permit a clearer comparison of effect sizes across categories, this approach was not feasible due to variations in the versions of the CADSS used by different author groups, which differ in both the included items and scores (16). SMDs were coded such that positive values reflect greater state dissociation (CADSS score) in the reference category than a control. Meta-analyses were supplemented with prediction intervals (PIs) when *k* ≥ 5 (21,22); PIs estimate the distribution of the effect in a future individual study with similar features. Heterogeneity of effect sizes was computed using *I*^2^ and τ^2^, where values exceeding 50% and 10% reflect moderate or greater heterogeneity, respectively. Publication bias was evaluated using funnel plots of SMDs against SEs and Egger’s bias test, where *p* < .05 reflects asymmetry (23); we computed revised SMDs correcting for asymmetry using the trim-and-fill method (24). Moderators of effect sizes were assessed using meta-regression analyses where there were at least 5 effect sizes in each category and at least 10 effect sizes within a category, respectively. Multiple preregistered analyses were not performed due to an insufficient number of effect sizes or insufficient information in original articles (Supplement). Analyses were performed in JASP (version 0.18.3, 2014; JASP Team), Jamovi (version 2.3.26.0; the Jamovi project), and MATLAB (version 2023a; The MathWorks, Inc.).

## Results

### Study Inclusion and Characteristics

A PRISMA diagram showing study selection is presented in Figure S1. A total of 123 articles met inclusion criteria, yielding 155 effect sizes (*n* = 6629) that could be included in our main analysis categories (see Supplemental Results for exclusions). After we excluded 9 outliers, the effect sizes included controlled comparisons of diagnostic categories (*k* = 32, *n* = 1729), psychological techniques (*k* = 50, *n* = 2400), or pharmacological agents (*k* = 64, *n* = 2563) (Table 1). The largest categories (*k*s ≥ 10) included posttraumatic stress disorder (PTSD), mirror gazing, trauma stimuli exposure, and ketamine. Methodological quality ratings and study details can be found in Table S2.

**Table 1:** Results of Meta-Analyses of State Dissociation Effects (CADSS Scores in Reference vs. Control) as a Function of Diagnostic Category, Psychological Technique, and Pharmacological Agent

Category	*k*	*n*	SMD	95% CI	PI	*z*	*p*	*I*^2^	τ^2^	FPA *p*	Outliers
Diagnostic Categories											
PTSD-DC	7	443	1.34	0.86 to 1.82	−0.23 to 2.91	5.44	<.001	78.77%	0.31	.013	0
PTSD	12	644	0.94	0.65 to 1.23	0.04 to 1.84	6.42	<.001	61.66%	0.14	.030	0
MDD	6	338	0.89	0.43 to 1.35	−0.63 to 2.41	3.82	<.001	75.27%	0.24	.77	0
SZ	3	146	0.86	0.51 to 1.21	−1.49 to 3.21	4.83	<.001	0%	0	.34	0
FND	4	158	0.59	−0.17 to 1.35	−2.84 to 4.02	1.52	.13	80.59%	0.48	.086	0

Psychological Techniques											
Mirror Gazing	12	392	0.94	0.52 to 1.35	−0.63 to 2.51	4.44	<.001	86.43%	0.45	<.001	0
Military Training	9	639	0.77	0.52 to 1.02	−0.07 to 1.61	6.10	<.001	80.1%	0.11	.37	1
Sleep Deprivation	3	110	0.56	0.26 to 0.86	−2.69 to 3.81	3.69	<.001	52.2%	0.04	.063	1
Trauma Stimuli	18	902	0.50	0.36 to 0.64	−0.01 to 1.00	6.94	<.001	63.68%	0.05	.15	1
Complementary Methods	3	232	0.41	0.14 to 0.67	−1.76 to 2.58	2.99	.003	22.29%	0.01	.23	0
Negative Affect Stimuli	5	125	0.16	−0.02 to 0.34	−0.15 to 0.47	1.79	.074	0%	0	.20	1

Pharmacological Agents											
Ketamine	47	1579	1.51	1.23 to 1.80	0.17 to 2.85	13.70	<.001	83.65%	0.42	<.001	4
Cannabis	4	139	1.40	0.96 to 1.83	−1.39 to 4.19	6.30	<.001	72.76%	0.37	.002	0
N_2_O	3	129	1.16	0.91 to 1.41	−0.53 to 2.85	9.00	<.001	0%	0	.42	0
Psychedelics	4	68	1.16	0.66 to 1.67	−0.91 to 3.23	4.50	<.001	63.03%	0.16	.34	1
Esketamine	6	648	0.94	0.55 to 1.33	−0.28 to 2.16	4.77	<.001	78.87%	0.15	.001	0

*k* indicates the number of included effect sizes (after removal of outliers); *I*^2^ and τ^2^ are heterogeneity statistics; and outliers indicates the number of outliers removed.

CADSS, Clinician-Administered Dissociative States Scale; FPA *p*, funnel plot asymmetry *p* value; FND, functional neurologic disorder; MDD, major depressive disorder; N_2_O, nitrous oxide; PI, prediction interval; PTSD, posttraumatic stress disorder; PTSD-DC, PTSD-dissociative and complex subtypes; SMD, standardized mean difference; SZ, schizophrenia.

### Meta-Analyses of Controlled Comparisons of State Dissociation in Diagnostic Categories

**Figure 1:**
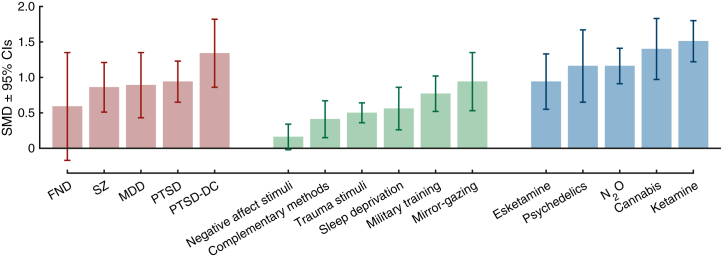
Results of meta-analyses of state dissociation (Clinician-Administered Dissociative States Scale scores) at baseline in diagnostic categories (red), in response to psychological interventions (green), and in response to pharmacological agents (blue). FND, functional neurological disorder; MDD, major depressive disorder; PTSD, posttraumatic stress disorder; PTSD-DC, PTSD dissociative and complex subtypes; SMD, standardized mean difference; SZ, schizophrenia.

**Figure 2:**
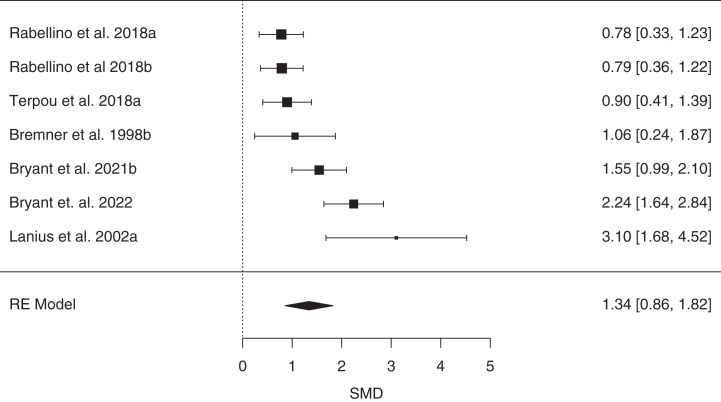
Forest plot of standardized mean differences (SMDs) (with 95% CIs) of baseline state dissociation (Clinician-Administered Dissociative States Scale scores) in posttraumatic stress disorder dissociative and complex subtypes relative to controls. Marker sizes reflect study weights with smaller markers denoting smaller study weights. RE, random effects.

The magnitude of state dissociation at baseline was examined in 5 diagnostic groups to provide a reference point for effect sizes for induced dissociative states (Table 1 and Figure 1). Dissociative states were significantly greater than nonclinical control participants in all groups except functional neurological disorder (FND). Baseline state dissociation was most pronounced in patients who met criteria for the dissociative and complex subtypes of PTSD (PTSD-DC) (SMD = 1.34) (Figure 2), followed by weaker, but still large, effects in PTSD, major depressive disorder (MDD), and schizophrenia (SZ), which showed comparable effect sizes (analyses of CADSS subscales were not possible due to an insufficient number of studies) (for forest plots, see the Supplement). PIs were only significant in PTSD, whereas moderate-to-large heterogeneity was observed in all groups except the SZ group.

### Meta-Analyses of Psychological Techniques for the Induction of Dissociative States

**Figure 3:**
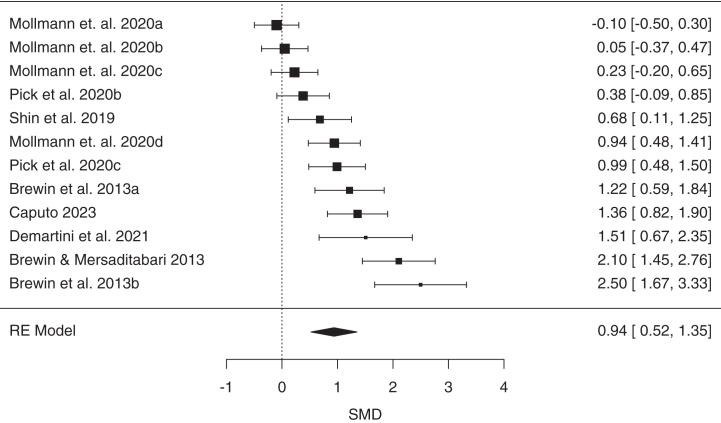
Forest plot of standardized mean differences (SMDs) (with 95% CIs) of induced state dissociation (Clinician-Administered Dissociative States Scale scores) in response to mirror gazing relative to a control condition. Marker sizes reflect study weights, with smaller markers denoting smaller study weights. RE, random effects.

All induction methods significantly increased state dissociation except negative affect stimuli exposure (Table 1 and Figure 1). Mirror gazing was associated with a large effect size (SMD = 0.94) (see Figure 3) followed by a large effect for military training, whereas sleep deprivation, trauma stimuli exposure, and complementary methods elicited moderate effects (for forest plots, see the Supplement). PIs were nonsignificant for all methods. Moderate-to-large heterogeneity in effect sizes was observed for mirror gazing, military training, and trauma stimuli exposure. Additional analyses suggested that the effects of mirror gazing were most pronounced for derealization (Table S3).

### Meta-Analyses of Pharmacological Induction of Dissociative States

**Figure 4:**
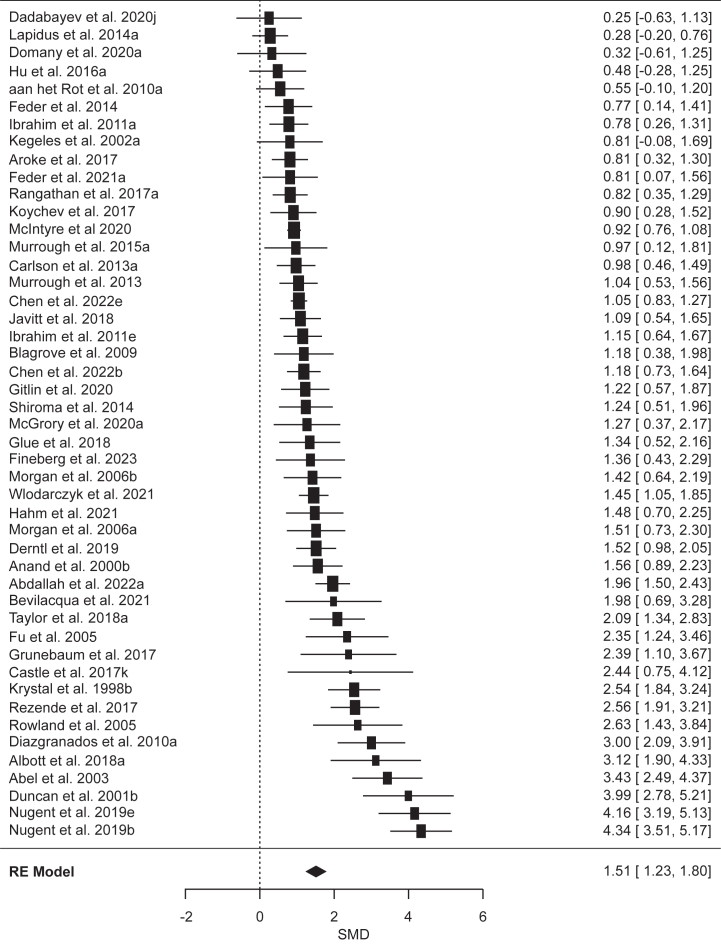
Forest plot of standardized mean differences (SMDs) (with 95% CIs) of induced state dissociation (Clinician-Administered Dissociative States Scale scores) in response to ketamine relative to a control condition. Marker sizes reflect study weights, with smaller markers denoting smaller study weights. RE, random effects.

The analyses of pharmacological agents revealed that all agents reliably induced dissociative states with large effect sizes (Table 1 and Figure 1) (for forest plots, see the Supplement). The largest effect sizes were observed for ketamine (Figure 4) and cannabis (SMDs > 1.35), with slightly weaker effects for N_2_O and psychedelics (SMDs = 1.16) and the weakest, although still large, effect for esketamine. PIs were significant only for ketamine, and moderate-to-large heterogeneity in effect sizes was observed for all agents except N_2_O. Analyses of subdimensions of dissociation yielded comparable results, although the effects were generally larger for derealization across all agents (see Table S3). Additional analyses suggested that the dissociative effects of ketamine were most pronounced during the first 30 minutes postinfusion (see Table S4).

### Publication Bias

Among diagnostic categories and psychological techniques, the effect sizes for PTSD-DC, PTSD, and mirror gazing showed significant evidence of funnel plot asymmetry (see Table 1 and the Supplement for funnel plots). By contrast, significant funnel plot asymmetry was observed for all pharmacological agents except N_2_O and psychedelics. These results are reflective of potential publication bias and suggest that effect size estimates for multiple categories may be inflated.

### Meta-Regressions

Meta-regression analyses were undertaken to examine whether variability in effect sizes across studies was attributable to different methodological features across studies including the induction method, administration method, sample, design, and other methodological features. Our first set of meta-regression analyses compared state dissociation effects across categories in cases where *k* ≥ 5 in each category (Table S5). Both mirror gazing and military training elicited larger increases in state dissociation than exposure to trauma and negative affect stimuli (ΔSMDs > 0.30), but the former two did not differ significantly. In contrast, no significant differences were found across diagnostic categories or pharmacological agents. Baseline dissociation in PTSD-DC was greater than induced dissociation for all induction methods (ΔSMDs > 0.50) except for mirror gazing and pharmacological agents. Baseline dissociation in PTSD and MDD was greater than induced effects from trauma and negative affective stimuli exposure but weaker than pharmacological induction effects. Finally, comparisons across categories indicated that induced dissociation was greater in response to ketamine than all psychological techniques (ΔSMDs > 0.50), whereas the response to esketamine was only greater relative to trauma and negative affective stimuli exposure.

With our final set of meta-regression analyses, we sought to clarify whether heterogeneity in effect sizes was associated with different methodological features (Table S6). Effect sizes were not significantly moderated by methodological quality or administration method (clinician/experimenter vs. self-report). Nonclinical samples showed a stronger dissociative response to ketamine than clinical samples (ΔSMD = 0.46), but the 2 groups did not differ significantly in other categories. Experimental design did not uniformly moderate effect sizes, with larger induction effects for between-groups designs and within-groups designs for mirror gazing and ketamine, respectively. Induction effects did not differ across different types of control conditions for psychological techniques, whereas among ketamine studies, effect sizes were greater in studies that used inert placebo controls than baseline or active drug controls but were not significantly moderated by dose or route of administration.

## Discussion

In this meta-analysis, we sought to quantify and compare baseline state dissociation effects in clinical samples and induced state dissociation effects in response to psychological techniques and pharmacological agents. Baseline state dissociation was elevated in multiple diagnostic categories relative to controls but was most pronounced in individuals with PTSD-DC. Among induction studies, multiple pharmacological agents elicited pronounced dissociative effects in clinical and nonclinical samples. Mirror gazing was the most robust psychological technique, closely approximating the dissociative effects of pharmacological agents. These results reinforce state dissociation as a prominent transdiagnostic symptom (2) and demonstrate that clinically significant dissociative states can be reliably induced using a range of methods (9,10).

Our analyses confirmed the presence of elevated baseline state dissociation across several diagnostic categories. Baseline dissociation was most pronounced in PTSD-DC and PTSD, although most studies did not distinguish between PTSD subgroups. Elevated state dissociation in these groups is broadly consistent with previous analyses of trait dissociation (2), although our results diverge from the latter analysis insofar as individuals with SZ and depressive disorders showed comparable, although weaker, dissociative effects than individuals with PTSD in our analysis. Moreover, whereas individuals with FND have been shown to display high levels of trait dissociation, comparable to those of individuals with PTSD (2,5), FND was characterized by only moderate levels of state dissociation in our analyses and was the only nonsignificant diagnostic category. This discrepancy is plausibly attributable to the small number of studies that included FND samples and the greatest heterogeneity among all diagnostic categories, likely driven by differential levels of dissociation in FND subgroups (5). Although state and trait dissociation are strongly associated, they should be distinguished in research and clinical practice, because state dissociation may indicate more severe psychopathology (25). These results reinforce the importance of measuring dissociation in different diagnostic categories and clinical contexts, particularly given that dissociation may predict broader symptomatology (5, 6, 7) and treatment outcomes (26).

Analyses of pharmacological agents revealed that 2 agents elicited state dissociation effects that were comparable to, or exceeded, baseline dissociation in individuals with PTSD-DC. The most pronounced effects were observed with ketamine and cannabis, with slightly weaker, although still large, effects in N_2_O, psychedelics, and esketamine. Additional analyses suggested that ketamine’s dissociative effects are greatest the first 50 minutes postinfusion and larger in nonclinical samples. Taken together, these results indicate that different types of pharmacological action can produce large dissociative effects. Accordingly, dissociative states may not be associated with the perturbation of a specific neurochemical system but rather with broader network-level increases in neural signal complexity and changes in intra- and internetwork connectivity that are shared across these agents (27,28) and potentially with clinical samples (29) [for a consideration of neurophysiological differences across some of these agents, see (30)]. For example, ketamine, N_2_O, and lysergic acid diethylamide (LSD) are all associated with aberrant functional connectivity in nodes of the default mode and dorsal attention networks (e.g., the precuneus and temporoparietal junction) (28), which may parallel atypical precuneus and temporoparietal volume and/or functional connectivity in individuals with high dissociation (31, 32, 33, 34). These effects may reflect disruptions in embodiment and multimodal integration that play a central role in experiences of depersonalization and derealization or distortions in features of subjective experience subserved by a broader posterior cortical hot zone, which has been hypothesized to play a critical role in supporting the subjective contents of consciousness (28). Continued research into these other compounds may also help advance research into pharmacotherapeutic agents for reducing dissociative symptomatology; for example, whereas N_2_O acts a partial agonist of opioid receptors (30), preliminary research suggests that opioid antagonists seem to reduce dissociative symptoms (35) [also see (36)].

Among psychological techniques for inducing dissociative states, mirror gazing was the only method that elicited dissociative effects comparable to those observed in diagnostic categories and with pharmacological agents. In particular, the magnitude of the dissociative response to mirror gazing was similar to baseline dissociation in PTSD (and larger than all categories except PTSD-DC) and induced dissociation in response to esketamine but was weaker than all other pharmacological agents. The neurocognitive substrates of mirror gazing remain largely unknown, but it may produce dissociative states, particularly depersonalization, through a partial decoupling of visual and cognitive self-referential processing (1,37). By contrast, stress induction methods used in military/survival training elicited weaker, although still large, effects that were larger than the moderate and nonsignificant effects observed for exposure to trauma stimuli and negative affect stimuli, respectively. The greater efficacy of the former is plausibly attributable to its status as a more uniform stressor than tasks that involve different types of stimulus presentation with variable effects across individuals. Techniques that target awareness and attention (sleep deprivation, complementary methods) also produced moderate dissociative effects, which is consistent with accumulating evidence for a link between sleep disturbances and dissociation (1). Although typically viewed as a consequence of stress (1,3), these results cumulatively indicate that dissociative states can be reliably induced through a variety of methods such as by modulating awareness, perception, and sleep and highlight the need for direct comparisons of these methods and their neurocognitive substrates (1,9).

The observed state dissociation effects have direct implications for the development of an experimental model of dissociation (38). The cumulative data point to the greater efficacy of mirror gazing than stress induction methods, given that it produces larger dissociative effects and is less likely to trigger adverse events (9,39). Our results also highlight ketamine, cannabis, and N_2_O as the most robust pharmacological agents for inducing dissociation; the latter is particularly well suited to experimental research given that its low blood solubility elicits rapid induction and termination effects (10,39). Although these results are not formally incompatible with the broad consensus that dissociative psychopathology is a consequence of developmental trauma (40), they underscore the need for direct comparisons between methods. Preliminary research suggests that script-driven imagery methods of inducing dissociation seem to be associated with activation patterns [e.g., greater amygdala activation (41)] that differ from those that involve pharmacological agents (28). Accordingly, further neurophysiological research that compares different methods is necessary to understand the extent to which these methods have overlapping and distinct neurocognitive substrates. Preliminary trends suggest that different pharmacological agents and mirror gazing produce greater derealization than depersonalization; further targeting these effects could be beneficial in elucidating the neural correlates of subdimensions of dissociation (42). Development of experimental models of dissociation will also require greater attention to the temporal dynamics of and dosing effects on state dissociation, which are poorly understood apart from ketamine. Our analyses suggest that clinical samples show weaker dissociative responses to ketamine, and previous research points to trait dissociation as a predictor of such responses (26); more attention to the sources of individual differences in response to induction methods is necessary. Finally, although our meta-analysis demonstrates that mirror gazing and multiple pharmacological agents can induce dissociative states that are large in magnitude and comparable to baseline dissociation in some clinical samples, further research is required to assess their clinical relevance in comparison to dissociative effects in diagnostic categories.

### Limitations

The principal limitations of this meta-analysis concern limited available data in specific categories and methodological weaknesses in the original studies. Many categories included a small number of effect sizes, thereby limiting the precision of our estimates and preventing us from examining sources of heterogeneity. Our choice to restrict our analyses to studies that used the CADSS facilitated comparisons across categories and ensured a good degree of phenomenological uniformity in response patterns but might have excluded important research with other validated instruments (16). In turn, it will be important for future empirical studies and meta-analyses to compare and contrast the CADSS with these other measures. Only a small proportion of studies reported CADSS subscale scores (e.g., depersonalization), thereby limiting our analyses of different subdimensions of state dissociation. It remains unclear whether this omission reflects publication bias, poor psychometric properties of specific subscales, or other factors, but further research into these subscales and their psychometric properties and discriminant validity is required. Only a small minority of studies included trait dissociation measures, which prevented us from assessing their value in predicting dissociation induction effects (26). State dissociation was alternately measured peri-induction (most pharmacological agents) or postinduction (most psychological techniques), which might have introduced different response biases that were not captured in our analyses. Relatedly, most of the original studies are potentially confounded by demand characteristics and potential placebo effects because participants are likely to have become unblinded to experimental conditions due to psychoactive effects (43). We planned to probe this in our preregistered analyses by examining the presence of suggestions for dissociative responses during procedures, but this information was not reliably reported and so could not be analyzed. Insofar as dissociation was typically measured as a secondary outcome or adverse event (13), these types of biases may be less pronounced than for psychedelic effects, but further consideration of this issue is warranted, such as through the use of active drug controls, stringent reporting of suggestion effects, and statistical corrections for unblinding effects (44).

Aside from ketamine, studies did not report state dissociation at multiple time points, thereby disenabling systematic analyses of peak dissociation effects. We were unable to examine the potential confounding effects of concurrent psychotropic medication in clinical samples. Except for ketamine, we were unable to examine the moderating impact of dose on state dissociation effects due to small sample sizes. Moreover, most studies reported ketamine (and other agent) doses in mg/kg, which does not account for individual differences in drug absorption, metabolism, distribution, and excretion (45), leading to variability in plasma concentrations and dissociative effects that could not be captured in our ketamine dose analyses. For this reason, our observation of a nonsignificant effect of ketamine dose on state dissociation should be treated with caution. Many of the agents that we analyzed elicit broader psychotomimetic effects (e.g., hallucinations) that could overshadow more subtle dissociative responses (10,12,46), thereby potentially limiting the measurement reliability of state dissociation (47).

### Conclusions

This meta-analysis confirmed that state dissociation is a transdiagnostic symptom present in multiple psychiatric conditions that can be reliably induced using different pharmacological agents as well as mirror gazing. These findings have direct implications for the experimental modeling of dissociation in controlled research, the search for neurophysiological markers of dissociation, and the assessment of adverse events and treatment outcomes in psychopharmacological interventions that involve NMDAR antagonists and classic psychedelics.

## Supplementary Material

Supplemental Methods, Analyses, Results, Figures S1–S30, Tables S1–S6

Key Resources Table
